# Research on the Hydrophobicity of Square Column Structures on Monocrystalline Silicon Fabricated Using Micro-Machining

**DOI:** 10.3390/mi10110763

**Published:** 2019-11-11

**Authors:** Ziyang Cao, Wenyu Ding, Zhenwu Ma, Bangfu Wang, Zhongwang Wang

**Affiliations:** 1 College of Mechanical Engineering, Suzhou University of Science and Technology, Suzhou 215009, China; luckie_d@126.com (W.D.); mazw@usts.edu.cn (Z.M.); sunboy02@126.com (B.W.); maxwang_zw@126.com (Z.W.); 2 Suzhou Key Laboratory of Precision and Efficient Machining Technology, Suzhou 215009, China

**Keywords:** prediction model, square column structure, hydrophobicity, micro-milling, molecular dynamics

## Abstract

The theoretical prediction models of contact angle were constructed by considering the interface free energy. Then, the square column structure on monocrystalline silicon was fabricated using micro-milling. The rationality of prediction models was validated by regulating the parameters of the square column. It should be mentioned that the whole construction process was facile and efficient. After processing, the hydrophobicity of monocrystalline silicon with the square column structure was improved. The static contact angle of the processed monocrystalline silicon reached 165.8° when the side length of the square column was 60 μm. In addition, the correctness of the prediction models was verified from the perspective of molecular dynamics. The prediction models of contact angle were of great value for the practical application.

## 1. Introduction

In recent years, a great deal of research has been carried out on the hydrophobicity of materials and remarkable progress has been achieved [1]. As a superhydrophobic material, the leg of the water strider has attracted wide attention and been used in various fields in recent decades. A hydrophobic surface refers to a surface that is not easily moistened by water droplets, and its contact angle with water is not less than 90° [2]. The value of the contact angle can reflect the wettability of a solid surface, i.e., the smaller the contact angle, the better the wettability of the solid surface. Silicon is a key material for microelectronics, optoelectronics, and sensors, and has been widely used in the manufacturing of sensors, solar cells, and many other optical components. The microstructure of the surface of monocrystalline silicon can significantly regulate the hydrophobicity of this material. The existence of hydrophobicity can greatly change the wear resistance and drag reduction of the material and achieve the ability of self-cleaning. When the droplets are placed on a hydrophobic surface, the dust and impurities on the surface will be carried away from the material together with the droplets. Thus, the self-cleaning function of a solar device can be realized by adding a hydrophobic layer [3,4].

Monocrystalline silicon is naturally hydrophobic. These silicon-based hydrophobic surfaces are usually obtained by constructing micro or nanostructures on silicon surfaces to reduce the surface energy. To achieve a higher hydrophobicity, the micro or nanostructure is required for the surface of the silicon-based material [5]. Various approaches have been developed to construct hydrophobic surfaces. Yang et al. [6] and Gurav et al. [7] developed a sol-gel method to prepare new coatings. Chen et al. [8] used a special electrodeposition processing method to prepare a superhydrophobic cathode surface on a copper substrate in an electrolytic solution containing NiCl_2_·6H_2_O, tetradecanoic acid and ethanol. Wang et al. [9] adopted an ion etching method to make microstructures for Si. Razavi et al. [10] used chemical vapor deposition to form a microstructure on the surface of copper sheets. Hu et al. [11] used laser to fabricate the titanium alloy to get a biomimetic superhydrophobic surface. Varlamova et al. [12] modified the surface properties of Si using laser-induced periodic surface structures. However, hydrophobic surfaces are mostly constructed using chemical processing and non-traditional processing, and the hydrophobic structure generated by these processing methods is random. In the context of microfabrication, micro-milling technology has gradually developed. Compared with laser processing, Electric discharge machining (EDM) and other micro-machining technologies, micro-milling technology can realize the construction of three-dimensional structures and large-area structure preparation better [13]. The controllability and repeatability of the micro-milling is also good. The structure prepared using micro-milling technology can be well matched with the structural variables of the theoretical model to achieve an accurate exploration of the correlation between the structure and wettability of the surface [14].

Furthermore, theoretical models corresponding to different structures have been established. Marmur et al. [15] analyzed the cylindrical, hemispherical, parabolic and cylindrical protrusions, and constructed the corresponding models. Wang et al. [16] established a theoretical model of an aluminum alloy surface with square column structures that were based on the roughness factor. However, based on the exploration of Gibbs free energy and structural parameters, there are only a few models that explore the hydrophobicity of square column structures or to predict the contact angle of the square column structure visually. Based on Gibbs free energy, Shi et al. [17] constructed prediction models of contact angle for polymethyl methacrylate (PMMA) and Ti_6_Al_4_V with convex structures. However, the square column structures were not mentioned.

In this work, by combining the parameters of micro-structure and interface free energy, reasonable theoretical models were established. The contact angle value could be directly predicted through the parameters of the structure in the models. Furthermore, micro-milling was selected to construct the structure to verify the theoretical models. The whole construction process was facile and efficient, without the requirement of any special devices. The constructed theoretical models could predict static contact angles very well. In addition, experiments showed that excellent hydrophobicity usually occurred when the side length of square column was small. Finally, a simulation of molecular dynamics was carried out to verify the trend of the contact angle at the molecular level. The method of fabricating hydrophobic structure on monocrystalline silicon proposed in this study provides a reference for large-scale industrial production.

## 2. Establishment of the Theoretical Model

### 2.1. Basic Parameters of the Model

**Figure 1:**
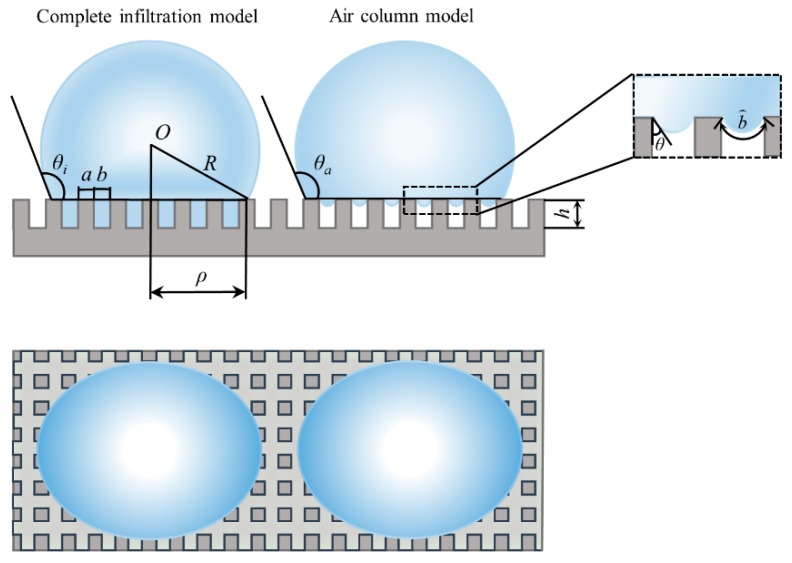
Diagram of the square column model.

Due to the variety of microstructures, existing models are aimed at predicting the contact angle of materials with rough surfaces. It is impossible to visually investigate the influence of microstructure parameters on the hydrophobic properties of materials. Thus, two kinds of models were built based on the wetting state. In this paper, the interface contact lines were straight lines. Then, according to the wetting phenomenon of droplets on the surface of the material, the constructed models were divided into a complete infiltration model and an air column model. Figure 1 shows the two kinds of models. *R* represents the radius of the contact surface of the droplet with the surface of the material, *a* represents the side length of square column, and *b* represents the spacing width of the square column. *h* is the height of square column, *ρ* represents the radius of the contact surface between the droplet and the solid. *θ_i_* and *θ_a_* are the contact angles under different wetting models, respectively. $\overset{⌢}{b}$ is the length of the gas-liquid contact line between the square column structures. *A_sl_* is the contact area of the solid-liquid interfaces, *A_lv_* is the contact area of the gas-liquid interfaces, and *A_sv_* is the contact area of the solid-gas interfaces.

The static contact angle was predicted using the model constructed based on the minimum Gibbs free energy. The Gibbs free energy can be obtained using the surface free energy and surface contact area:

(1) $$G\;=\;\gamma_{sl}A_{sl}+\gamma_{lv}A_{lv}+\gamma_{sv}A_{sv},$$

where *G* is the Gibbs free energy; *γ_sl_*, *γ_lv_*, and *γ_sv_* are the interfacial free energy of the solid-liquid, gas-liquid, and solid-gas interfaces, respectively; and *A_sl_, A_lv_*, and *A_sv_* are the contact area of the solid-liquid, gas-liquid, and solid-gas interfaces, respectively.

The Gibbs free energy will be minimized when the droplet reaches equilibrium on the surface of the material. Thus, *dG* = 0.

The value of surface tension is equal to the value of the surface free energy. By substituting this into Equation (1) and differentiating, this gives:

(2) $$\sigma_{sl}dA_{sl}+\sigma_{lv}dA_{lv}+\sigma_{sv}dA_{sv}=0$$

Based on the parameters of the constructed structure, the number of square column structures below the droplet can be expressed as Equation (3):

(3) $$N=\frac{\pi \rho^{2}}{{\left(2\rho \right)}^{2}}\cdot {\left(\frac{2\rho }{a+b}\right)}^{2}=\frac{\pi \rho^{2}}{{\left(a+b\right)}^{2}}.$$

### 2.2. Establishment of the Square Column Model

According to the exploration of the square column structure shown in Figure 1, the contact areas between the three phases were represented by the structural parameters, which are shown in Equations (4)–(6), respectively:

(4) $$A_{sl}=\pi \rho^{2}+N\cdot 4ah=\pi \rho^{2}+\frac{4ah\pi \rho^{2}}{{\left(a+b\right)}^{2}},$$

(5) $$A_{lv}=\int_{0}^{\theta_{i}}2\pi \rho Rd\theta =\frac{2\pi \rho^{2}}{1+cos\theta_{i}},$$

(6) $$A_{sv}=0.$$

By substituting these contact areas and Equation (2), the droplet balance equation $\sigma_{sv}=\sigma_{sl}+\sigma_{lv}cos\theta$ can be expressed as Equation (7), where *θ* is the intrinsic contact angle of monocrystalline silicon:

(7) $$cos\theta =\frac{dA_{lv}}{dA_{sl}}=\frac{{\left(a+b\right)}^{2}}{{\left(a+b\right)}^{2}+4ah}\cdot cos\theta_{i}$$

For the droplet infiltrate structure, the contact angle can be expressed as Equation (8), as follows:

(8) $$cos\theta_{i}=\left[1+\frac{4ah}{{\left(a+b\right)}^{2}}\right]cos\theta$$

In contrast, the contact area of the air column model is different from that of complete infiltration model. As shown in Figure 1, it looks like there is air between the square column structure and droplets in the air column model. For the air column model, the surface curvature resulted in an over-pressure in the liquid relative to the exterior pressure [18,19]. As a result, the gas-liquid contact line between the structures was not always a straight line.

The length of the gas-liquid contact line between square column structures is:

(9) $$\overset{⌢}{b}=\frac{b}{2sin(\theta +\delta )}\cdot \left(2\pi -2\theta -2\delta \right),$$

where *δ* is the angle between the square column and the plane. In this study, *δ* = π/2. The ratio of the gas-liquid contact line length to the spacing width of square column structure is:

(10) $$\frac{\overset{⌢}{b}}{b}=\frac{\frac{b}{2cos\theta }\cdot \left(\pi -2\theta \right)}{b}=\frac{\pi -2\theta }{2cos\theta }\approx 1$$

Thus, $\overset{⌢}{b}$ *≈ b*, which means the gas-liquid contact lines between the square column structures were approximately the line segment parallel to the boss.

Combining the wet state of the droplet on the structure and the structural parameters of the microstructure, the contact areas between the three phases were expressed as Equations (11)–(13), respectively:

(11) $$A_{sl}=N\cdot a^{2}=\frac{a^{2}\pi \rho^{2}}{{\left(a+b\right)}^{2}}$$

(12) $$A_{lv}=\int_{0}^{\theta_{a}}2\pi \rho Rd\theta +\pi \rho^{2}-\frac{a^{2}\pi \rho^{2}}{{\left(a+b\right)}^{2}}=\frac{2\pi \rho^{2}}{1+cos\theta_{a}}+\pi \rho^{2}-\frac{a^{2}\pi \rho^{2}}{{\left(a+b\right)}^{2}}$$

(13) $$A_{sv}=A_{total}-A_{sl}$$

Based on the contact areas and Equation (2), the droplet balance equation can be expressed as Equation (14):

(14) $$cos\theta =\frac{dA_{lv}}{dA_{sl}}=\frac{{\left(a+b\right)}^{2}}{a^{2}}\cdot cos\theta_{a}+\frac{{\left(a+b\right)}^{2}}{a^{2}}-1$$

The relationship between the contact angle and geometric parameters under the air column model can be obtained using Equation (15):

(15) $$cos\theta_{a}=\frac{a^{2}}{{\left(a+b\right)}^{2}}(cos\theta +1)-1$$

**Figure 2:**
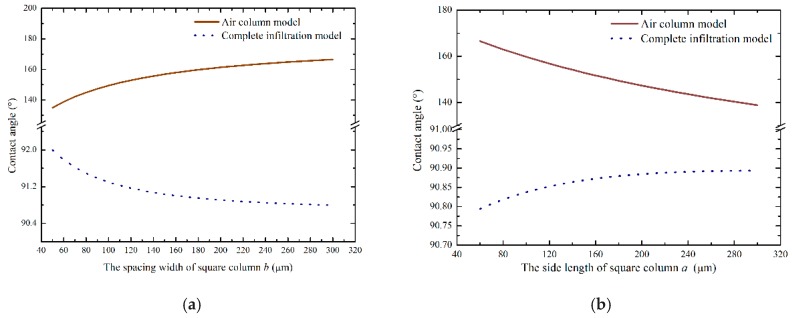
Correlation between the contact angle and structural parameters: (**a**) the spacing width of square column and (**b**) the side length of the square column.

Through Equations (8) and (15), the correlation between the contact angle and structural parameters were obtained using MATLAB (ver. Matlab 2016, MathWorks, Natick, MA, USA). Figure 2 shows the relationship between the contact angle and the structural parameters. According to Figure 2a, it can be seen that as the spacing width of the square column increased, the contact angle of the air column model gradually increased. When *b* = 300 μm, the contact angle was larger than for smaller *b* values. However, the trend of the contact angle predicted using the complete infiltration model was opposite to that of the air column model. Figure 2b shows the curve showing the relationship between the contact angle and the side length of the square column when *b* = 300 μm and *h* = 100 μm.

From Figure 2b, it can be seen that the changes in structural parameters had an effect on the hydrophobicity of monocrystalline silicon containing microstructures. The hydrophobicity of the air column model gradually increased as the length of the square column decreased. In contrast, the contact angle predicted using the complete infiltration model was larger as the side length of the square column increased.

## 3. Experimental and Simulation Verification

### 3.1. Experimental Verification

Experiments were carried out to verify the contact angle predicted by the prediction models. In this paper, the experimental samples were monocrystalline silicon wafers (1 0 0, Shunsheng, Ningbo, China). According to the physical properties of the monocrystalline silicon, 1 0 0 crystal planes were selected and the surface was polished. The diameter parameter of these samples was 25 mm, and the thickness was 400 μm [20,21]. All of these monocrystalline silicon samples were cleaned using an ultrasonic cleaner (JP-010T, Skymen, Shenzhen China) containing ethanol and a 10% HF aqueous treatment to remove the burrs and oxide grown on the surface of the samples. Then, the metallographic tapes were stuck on the surface of these nitrogen-dried samples to isolate it from the air, ensuring accurate measurements and processing. The workpieces were machined using a double-edged micro-milling cutter (NTS-Cutting, Natasi, Dongguan China). The machining system used in this paper was a high-speed milling center. The tool selected was a diamond coated micro-milling cutter with a diameter of 0.2 mm. The spindle speed was 48,000 r/min and the feed speed was 6 mm/min during experiments [22]. Six specimens were processed with 15 grooves distributed on the intermediate surface of the workpieces to form a square column structure.

**Figure 3:**
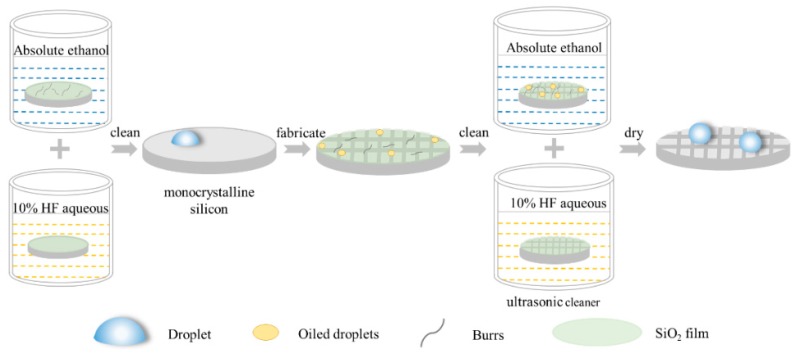
The processing flow chart for monocrystalline silicon.

All processed samples were cleaned with an ethanol-containing ultrasonic cleaner for 5 min to remove the burrs and impurities remaining on the surface and between the structures of the monocrystalline silicon. After the impurities were cleaned, the prepared 10% HF aqueous solution was placed in the ultrasonic cleaner to remove the oxide grown on the surface of samples. Finally, the surface of the specimens was dried using nitrogen for a follow-up analysis. The apparent morphology of samples was observed with a VHX-5000 Superhigh magnification lens zoom 3D microscope (VHX-5000, Keyence, Osaka, Japan). The static contact angles of these specimens were measured using an optical contact angle measuring instrument (OCA, DataPhysics, Filderstadt, Germany). It was necessary to measure the contact angle of each specimen five times to ensure the reliability of the measured data. Furthermore, a fixed area was selected as the measuring point of the contact angle to ensure accurate experimental data. The processing flow chart for creating monocrystalline silicon with square column structures is shown in Figure 3.

The purpose of this experiment was to verify the hydrophobicity of silicon surfaces containing square column microstructures by changing the side length of the square column.

In the experiment, the side length of the square column *a* was a variable, while the spacing width *b* and the height *h* of the square column were constant, where *b* = 300 μm and *h* = 100 μm. 

### 3.2. Simulation Verification

The state of the water droplets with different geometric surfaces was studied from the perspective of molecular dynamics. In this paper, the droplets were simulated using an SPC water model. The water-water and Si-water interactions in this paper were described using the Lennard-Jones (LJ) potential [23]:

(16) $$cos\theta_{a}=\frac{a^{2}}{{\left(a+b\right)}^{2}}(cos\theta +1)-1$$

The Si-water potential was obtained using the Lorentz-Berthelot rule:

(17) $$\varepsilon_{\mathrm{sw}}=\sqrt{\varepsilon_{s}\times \varepsilon_{w}}$$

(18) $$\sigma_{\mathrm{sw}}=\frac{\sigma_{s}+\sigma_{w}}{2}$$

**Figure 4:**
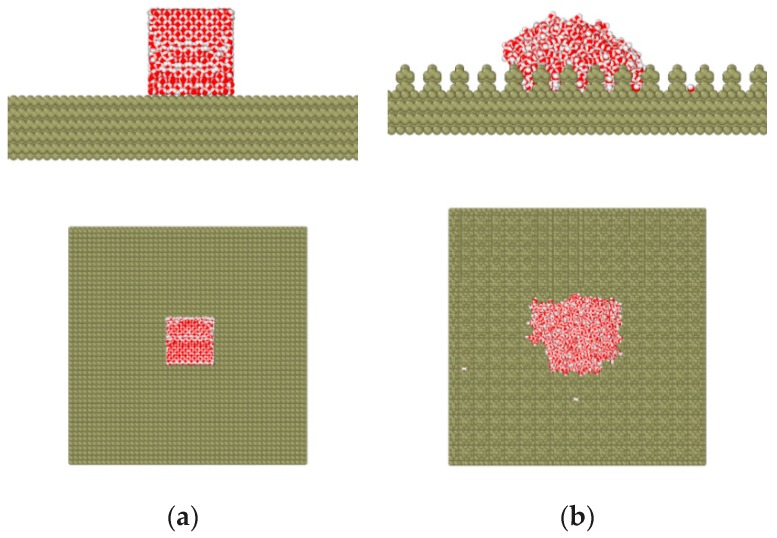
The schematics of the simulation model: (**a**) initial model and (**b**) model after simulation.

The simulation was carried out at 298 K. The Nose-Hoover temperature coupling algorithm was adopted in this paper. Figure 4 shows the schematics of the simulation model. The square column structure was obtained by removing the cells of the smooth structure [24].

**Figure 5:**
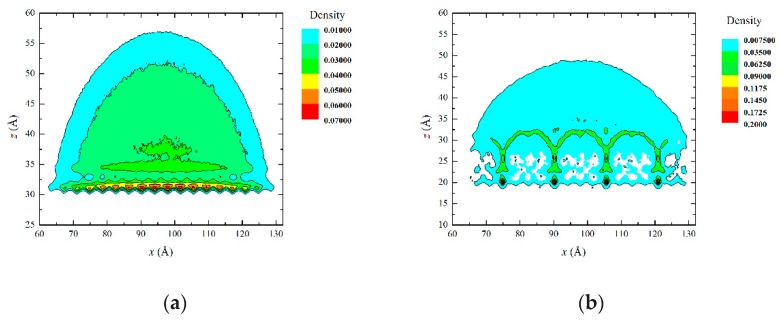
Density profile of a water droplet: (**a**) initial model and (**b**) model after simulation.

Figure 5 shows the density profile of the water droplet. In the density profile, the edge contour was extracted and the contact angle was derived at the solid-liquid boundary.

## 4. Results and Discussion

**Figure 6:**
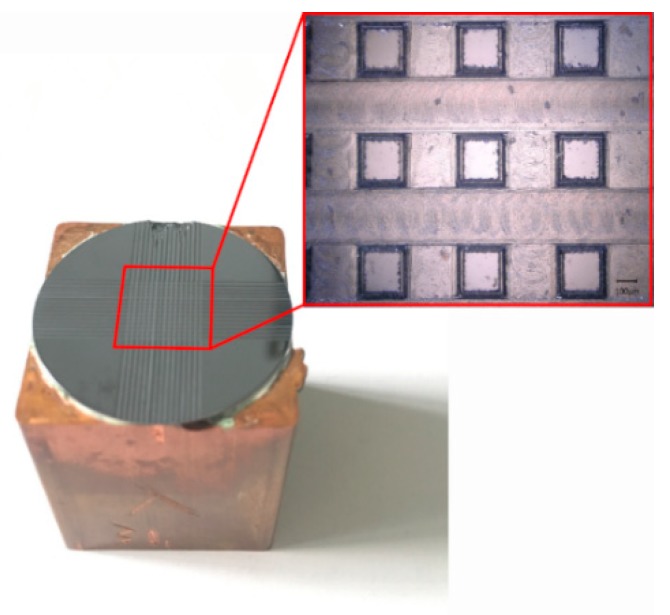
Surface diagram of the processed square column structure.

**Figure 7:**
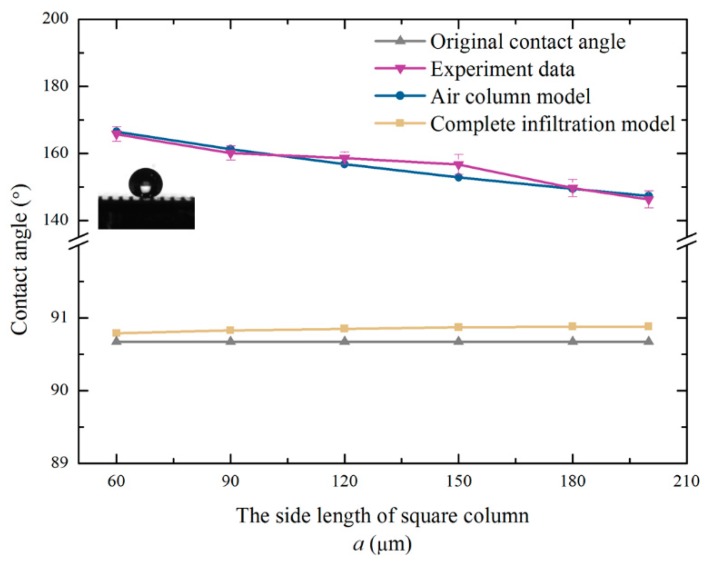
Variation curve of the contact angle between measurement and theory.

According to multiple measurements, the average contact angle of the unprocessed monocrystalline silicon was 90.67°. The monocrystalline silicon was naturally hydrophobic. Figure 6 shows the structure diagram of the samples after machining. The theoretical value of the contact angle was calculated according to Equations (8) and (15). The experimentally measured value and predicted value of the contact angle are shown in Table 1. Figure 7 is plotted based on the data in Table 1.

It is clear that the contact angles of the six specimens were all improved significantly. The hydrophobicity of the square column structure was dramatic. Furthermore, the air column model was more reliable than the complete infiltration model. Since the solid, liquid, and gas phases were in equilibrium, air was always present below the droplets. The boss constrained the droplet from all directions. Due to the high interfacial tension between the solid and liquid, the droplets were constrained, which caused an increase in the contact angle. The fluctuation between the predicted value and the measured value of the contact angle was very small. The reasonability of the prediction models was verified via the experiments. 

**Figure 8:**
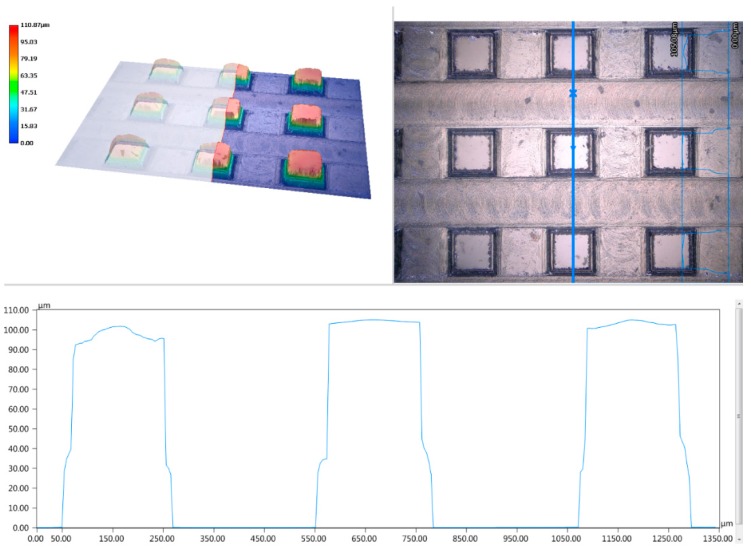
3D microstructure topography of the square column.

According to Figure 8, it can be seen that it was the cracking during processing that caused the slight fluctuation of the contact angle. Monocrystalline silicon is easier to curl during the milling process. Due to the large tensile stress of monocrystalline silicon, cracks and breakages occurred easily such that the measured contact angle was slightly larger than the theoretical value. Overall, the prediction models could calculate the contact angle of the square column accurately.

**Figure 9:**
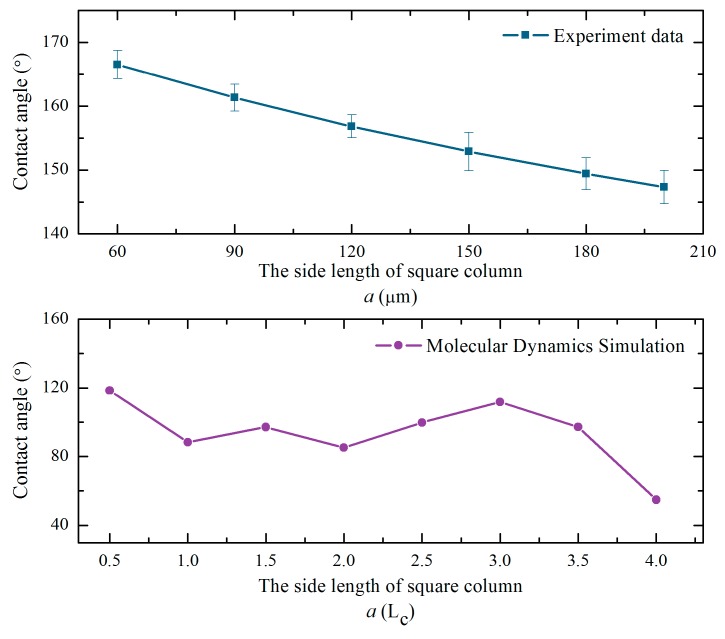
Variation curves of simulated and measured contact angle.

Figure 9 shows the contrast curve of the trend of the simulation data and experimental data. *L_C_* is the lattice constant of monocrystalline silicon. It can be seen that the trend of the simulated and measured values of the contact angle are the same. This indicates the correctness and reliability of the prediction model. It is clear that a smaller side length of the square column resulted in better hydrophobicity of solid surface in practical applications. The prediction models played a guiding role on the construction of the hydrophobic surface of monocrystalline silicon.

## 5. Conclusions

In this paper, the prediction models of contact angle were constructed by considering the interface free energy on the basis of micro-milling. Owing to the square column structure constructed using micro-milling, the surface could achieve a stable and effective hydrophobicity. There was a great match between experimental values and theoretical values, which proved the correctness and rationality of the prediction model. Particularly, the geometric parameters of the structure had a great influence on the contact angle. The smaller the side length of the square column, the larger the contact angle was, and the stronger the hydrophobicity of the material. Furthermore, the rationality of the experimental data trend was verified at the level of molecular dynamics, which ensured the correctness of the prediction model. Overall, it was clear that the prediction models played a guiding role in the construction of the hydrophobic surface on monocrystalline silicon. This study provided a reference for the large-scale industrial production of monocrystalline silicon with hydrophobic surface.

## Figures and Tables

**Table 1:** The theoretical and experimental values of contact angle.

Sample Number	Side Length of Square Column *a* (μm)	Complete Infiltration Model *θ_i_*(°)	Air Column Model *θ_a_*(°)	Experiment *θ*(°)
1	60	90.79	166.54	165.8 ± 2.2
2	90	90.83	161.33	160.1 ± 2.1
3	120	90.85	156.83	158.6 ± 1.8
4	150	90.87	152.89	156.7 ± 3.0
5	180	90.88	149.43	149.7 ± 2.5
6	200	90.88	147.34	146.3 ± 2.6
